# Integration of MRI radiomics with immune profiling to predict response to CDK6 inhibitors in high-grade glioma

**DOI:** 10.3389/fimmu.2026.1735070

**Published:** 2026-09-15

**Authors:** Jianfang Wang, Peng Li, Jinhu Li, Xiaolan She, Xiaodong Liu, Wang Miao, Xiaofeng Liu, Xufeng Liu

**Affiliations:** 1 Department of Neurosurgery, The Fifth People’s Hospital of Datong, Datong, Shanxi, China; 2 Department of Geriatrics, Beijing Hospital, National Center of Gerontology, Institute of Geriatric Medicine, Chinese Academy of Medical Sciences, Beijing, China; 3 Department of Neurosurgery, The First Hospital of Shanxi Medical University, Taiyuan, Shanxi, China; 4 Department of Neurology, The Fifth People’s Hospital of Datong, Datong, Shanxi, China

**Keywords:** CDK6 inhibitor, high-grade glioma, immuno-profiling, precision oncology, predictive biomarker, progression-free survival, radiomics

## Abstract

**Background:**

Predictive biomarkers are needed to identify patients with high-grade glioma who benefit from cyclin-dependent kinase 6 (CDK6) inhibition. We hypothesized that an integrated Radiomics–Immune Score (R/I-Score), combining MRI-derived tumor heterogeneity with peripheral immune profiling, would predict progression-free survival (PFS) benefit from CDK6 inhibitor–containing therapy (CDK6i).

**Methods:**

We conducted a two-center, retrospective cohort study utilizing longitudinal data from departmental neuro-oncology registries. The final study population comprised 116 adult patients with measurable disease who had undergone baseline contrast-enhanced MRI within 21 days prior to treatment initiation. To ensure methodological rigor and internal consistency, we adopted a two-stage study design. In the first stage (Biological Modeling), the total cohort (*N* = 116) was partitioned into a training set (*n* = 81) and an independent testing set (*n* = 35) to develop and validate the R/I-Score. In the second stage (Clinical Validation), we addressed the selection bias inherent in observational data by performing 1:2 Propensity Score Matching (PSM). This yielded a balanced analytical cohort of 108 patients (36 treated with CDK6i and 72 concurrent contro\ls). The primary endpoint was the treatment-by-biomarker interaction for progression-free survival (PFS), specifically assessing the R/I-Score’s ability to stratify therapeutic responders.

**Results:**

In the overall matched cohort (*N* = 108), patients treated with CDK6i exhibited numerical but statistically non-significant trends toward improved efficacy compared to concurrent controls in terms of median PFS (4.8 *vs*. 3.5 months; HR 0.72, *P* = 0.091), objective response rate (19.4% *vs*. 9.7%; *P* = 0.180), and disease control rate (44.4% *vs*. 30.6%; *P* = 0.165). The Combined R/I-Clinical Model demonstrated exceptional diagnostic performance in the independent testing cohort (*N* = 108), achieving a Treatment-Effect AUC of 0.97 and excellent calibration. In the PSM efficacy analysis, the R/I-Score successfully identified a distinct responder subpopulation: patients with a High R/I-Score derived significant survival benefit from CDK6i compared to concurrent controls (HR 0.48; 95% CI 0.25–0.91; *P* = 0.008), whereas those with a Low R/I-Score did not (HR 1.05; 95% CI 0.58–1.92). The interaction between the R/I-Score and treatment was statistically significant (*P_interaction_* = 0.012). Furthermore, High R/I-Score patients achieved superior objective response rates (33.3% *vs.* 5.6%) and sustained treatment duration (median 6 *vs.* 3 cycles) without increased toxicity (Grade ≥3 AEs: *P*>0.05). Biological interpretability analysis revealed that the score was driven by tumor morphological complexity and NK/CD8+ T-cell infiltration.

**Conclusions:**

The multi-modal R/I-Score serves as a robust, non-invasive predictive biomarker for CDK6i therapy in GBM. By selecting patients based on specific radiomic-immune phenotypes, this tool facilitates precision oncology, maximizing therapeutic efficacy while avoiding futile treatment in non-responders.

## 1 Introduction

High-grade gliomas (HGG) remain uniformly lethal despite incremental advances in surgery, radiochemotherapy, and supportive care, with outcome heterogeneity that persists even after adoption of molecularly integrated diagnostics in the 2021 WHO classification (1). Across real-world series and contemporary reviews, median survival for IDH-wildtype glioblastoma scarcely exceeds 15–20 months and varies widely between patients with ostensibly similar clinicopathologic profiles, underscoring the limitations of biopsy-based biomarkers to capture spatial heterogeneity and the immunosuppressive tumor microenvironment (TME) that drives progression and treatment failure (2–4). A parallel transformation in neuro-oncologic imaging has elevated MRI from descriptive readouts to quantitative radiomics/radiogenomics, extracting high-dimensional features (shape, intensity, texture, and habitat-level metrics) that noninvasively proxy tumor biology and correlate with survival, angiogenesis, hypoxia, and immune infiltration (5, 6). In HGG specifically, radiomics has been linked to macrophage abundance and immune gene programs, offering a translational bridge between image phenotypes and glioma immunobiology (7, 8). At the same time, the cyclin-dependent kinase 4/6 (CDK4/6) axis remains a central driver of glioma proliferation and therapeutic resistance; comprehensive preclinical and translational syntheses in the last five years have clarified canonical (RB/E2F-mediated) and non-canonical (transcriptional/epigenetic) roles of CDK6 in tumor progression and TME crosstalk, and they support biomarker-guided testing of CDK4/6 inhibitors in brain tumors (9–11). Yet, clinical experience suggests heterogeneous benefit to CDK6 inhibitors, with response modulated by pathway integrity (RB competence), lineage programs, and myeloid/NK-cell states—factors that are variably sampled by tissue assays but may be measurable by imaging and bulk-transcriptomic deconvolution (3, 4, 10–12). Beyond RB/E2F-driven cell-cycle control, CDK6 exhibits non-canonical functions in transcriptional and epigenetic regulation that can influence stromal and immune crosstalk. Emerging evidence across solid tumors suggests CDK4/6–pathway dependency interacts with immune context—particularly myeloid inflammatory programs and NK/CD8 effector competence—helping explain heterogeneous benefit from CDK4/6 blockade. These considerations provide a mechanistic rationale for integrating MRI-derived tumor habitat/heterogeneity signatures with immune profiling to predict CDK6i benefit in HGG.

These converging observations motivate an integrated, noninvasive framework to predict response to CDK6 inhibitors by combining pre-treatment MRI radiomics with immune profiling. Methodologically, quantitative radiomics from CE-T1WI (and, when available, T2/FLAIR, DWI/DSC) can summarize whole-tumor phenotype and peritumoral habitats, whereas digital cytometry (e.g., CIBERSORTx) applied to institutional RNA-seq or immunohistochemical surrogates estimates macrophage (M0/M2), NK, CD8 T-cell, and dendritic-cell fractions that shape drug sensitivity (5–8, 12, 13). Recent single-cell atlases and spatially informed studies have highlighted dominant myeloid programs, T-cell dysfunction, and interpatient variation that plausibly condition response to CDK6 blockade and rational combinations (e.g., CDK6 inhibition with CSF1R targeting or NK-cell–based strategies) (3, 4, 11). Critically, emerging radiogenomic work indicates that radiomics can predict CDK6 expression and RB-pathway activity, connecting macro-scale imaging features to cell-cycle signaling (8, 14). Within an evaluative design aligned to modern causal standards—namely, target-trial emulation using propensity-based overlap weighting and marginal structural models—an integrated radiomics-immune score could quantify heterogeneous treatment effects (HTE), provide decision-curve demonstrable clinical utility, and support biomarker-enriched trials (2, 6, 8–11, 13). Taken together, recent advances in glioma classification, radiomics, and immunogenomics create the methodological and biological rationale to develop and validate a multimodal predictor of benefit from CDK6 inhibition in HGG.

Our group has curated an institutional HGG registry harmonizing pre-treatment MRI, transcriptomic data, and adjudicated outcomes, and has previously demonstrated the feasibility of linking radiomic signatures to immune infiltration and prognosis in gliomas, including signatures associated with macrophage abundance and CDK6-related biology (7, 8, 14). Building on this foundation, the present study is designed to (i) develop and externally validate a pre-specified Radiomics–Immune Score (R/I-Score) that predicts clinical benefit from CDK6 inhibitors at first/second recurrence; (ii) quantify treatment-by-biomarker interaction for progression-free survival and response within a target-trial emulation, controlling confounding with overlap weighting; and (iii) provide biological anchoring and explainability, relating salient imaging habitats and immune states (macrophage/NK balance, RB/E2F program activity) to individualized treatment effect estimates. We hypothesize that MRI-derived habitat and texture features capture spatial correlates of CDK6 pathway activity, that immune composition refines these predictions by representing TME constraints on drug efficacy, and that the integrated R/I-Score will improve risk stratification and net clinical benefit beyond clinicopathologic models, thereby informing rational patient selection and combination strategies for CDK6-directed therapy in HGG.

## 2 Materials and methods

### 2.1 Patient cohort and data collection

This retrospective study included patients diagnosed with high-grade glioma (grade III or IV) at *The Fifth People’s Hospital of Datong and The First Hospital of Shanxi Medical University* between 2010 and 2020.

The initial screen identified 204 adult patients with histopathologically confirmed high-grade glioma (WHO grade 3 or 4). Eligibility for the final effectiveness analysis was contingent upon satisfying three domains of data completeness: (1) Imaging: availability of high-quality preoperative contrast-enhanced T1-weighted (CE-T1WI) MRI acquired within 21 days prior to the initiation of the index line of therapy; (2) Clinical: measurable disease according to Response Assessment in Neuro-Oncology (RANO) criteria and documented survival outcomes; and (3) Biological: available tumor tissue for transcriptomic profiling and peripheral blood markers for immune scoring. A hierarchical exclusion pipeline was applied to the initial cohort (N = 204). At the first screening stage, 56 patients were excluded due to insufficient imaging latency (>21 days, n=28), non-measurable disease (n=10), or incomplete clinical follow-up (n=18). At the second biological and pharmacological screening stage, a further 32 patients were excluded to prevent confounding, specifically: prior exposure to CDK4/6 inhibitors (n=12), concurrent use of potent immune-activating agents (n=8), or quality-control failure in immune data processing (n=12). A total of 116 eligible patients were included. For the purpose of radiomics model construction, this cohort was randomly partitioned into a training set (70%, n = 81) and an independent testing set (30%, n = 35). For the evaluation of clinical treatment effects, to mitigate selection bias inherent in retrospective data, we performed 1:2 PSM. This process yielded a balanced final analytic cohort comprising 36 patients in the CDK6i group and 72 patients in the Control group (Total *N_matched_* = 108).

Clinical variables such as age at diagnosis, sex, histologic subtype, tumor grade, and key molecular markers (IDH mutation status, 1p/19q codeletion, etc.) were recorded from pathology reports. CDK6 inhibitor therapy was defined as the administration of either Abemaciclib (150 mg orally twice daily on a continuous schedule) or Palbociclib (125 mg orally once daily for 21 days followed by 7 days off). All dosages were adjusted as clinically indicated for hematologic tolerability. Regimens were classified as ‘CDK6i-containing’ if the agent was administered for a minimum of one complete cycle (21–28 days), regardless of concurrent administration of alkylating agents (e.g., temozolomide) or anti-angiogenic therapy (bevacizumab). Overall survival (OS) was defined as the interval from initial surgery to death or last follow-up. Patients alive at last follow-up were censored. All patients provided informed consent for tissue banking and data use under a protocol approved by the Institutional Review Board of The Fifth People's Hospital of Datong (Approval No.: D2021-03). 

### 2.2 Imaging acquisition

Preoperative MRI was performed on 1.5T/3.0T scanners as per standard brain tumor protocols. For all patients, we obtained a contrast-enhanced T1-weighted image (CE-T1WI), which is routinely used to delineate tumor enhancement and blood–brain barrier breakdown. The axial CE-T1WI (post-gadolinium) was selected for radiomic analysis because it provides clear tumor delineation and is widely available. In addition, for a subset of patients, T2-weighted and FLAIR sequences were available but were not uniformly used in this analysis. Imaging parameters (field-of-view, slice thickness ~1 mm, matrix, etc.) were consistent across scans or adjusted in preprocessing to a common voxel size (**Supplementary Data**).

### 2.3 Tumor segmentation

Tumors were segmented on CE-T1WI scans to define the region of interest for feature extraction. An experienced neuroradiologist (blinded to outcomes) manually outlined the contrast-enhancing tumor region (excluding obvious necrosis or hemorrhage) on each slice. Segmentation was performed using ITK-SNAP software, generating a 3D binary mask of the tumor volume. To assess segmentation reliability, a second rater independently segmented 30 random cases; an intraclass correlation coefficient (ICC) was computed for volumetric agreement, yielding ICC > 0.85, indicating excellent consistency.

### 2.4 Radiomic feature extraction and selection

Radiomic features were extracted from each tumor region using the open-source PyRadiomics toolkit implemented in Python. We extracted a total of 107 quantitative features per tumor, encompassing multiple categories: 14 shape features (e.g. volume, surface area, sphericity), 18 first-order intensity statistics (histogram-derived metrics such as mean, median, kurtosis), and 75 texture features describing intra-tumoral heterogeneity (Gray Level Co-occurrence Matrix, Gray Level Size Zone Matrix, Gray Level Run Length Matrix, neighboring gray tone difference matrix, etc.). All images were preprocessed with voxel intensity normalization and resampling to a uniform resolution (1×1×1 mm) to minimize scanner variability. We applied image discretization with a fixed bin width for texture calculations, per IBSI (Image Biomarker Standardization Initiative) guidelines.

To ensure feature robustness, we evaluated intra-observer reproducibility for the radiomic features. The segmentation process was repeated on 20 cases by the same rater one month apart, and features with an ICC < 0.75 between the two extractions were excluded. This left 100+ stable features for analysis. Prior to modeling, all features were z-score normalized. Given the high dimensionality and multicollinearity of radiomic features relative to sample size, we performed dimensionality reduction via the Least Absolute Shrinkage and Selection Operator (LASSO) method. LASSO logistic regression with 10-fold cross-validation was used on the training cohort (see below) to select a parsimonious set of features most predictive of CDK6 expression. The optimal LASSO model (minimizing cross-validation error) retained three non-zero weight features, which were then used to construct the radiomics model. In our dataset, the optimal LASSO model retained seven non-zero weight features, including shape descriptors (Surface Area, Surface-to-Volume Ratio), first-order statistics (Kurtosis), and texture metrics (Entropy, LRHGLE, Coarseness, LAHGLE).

### 2.5 Gene expression and immune infiltration analysis

Tumor gene expression data were obtained from our institutional genomic database. For each patient, frozen tumor specimens (from surgery) underwent RNA sequencing (Illumina platform) and bioinformatic processing. Read counts were normalized to transcripts per million (TPM) and log^2^-transformed. We specifically extracted CDK6 gene expression values for each tumor. In addition, we performed CIBERSORTx immune cell deconvolution on the bulk RNA-seq data to estimate the relative fractions of various immune cell types within the tumor tissue. CIBERSORTx was run using the LM22 reference signature, which quantifies 22 immune subsets (including T cell subpopulations, B cells, NK cells, macrophage subtypes, dendritic cells, etc.) from bulk transcriptomic profiles. Cases with output CIBERSORT *p* < 0.05 were considered to have an adequate deconvolution. We focused on major immune populations such as M0/M1/M2 macrophages, T lymphocytes (CD8^+^ T cells, follicular helper T cells, regulatory T cells), B lymphocytes, NK cells, monocytes, and dendritic cells.

For certain analyses, CDK6 expression was dichotomized into “CDK6-high” *vs*. “CDK6-low” groups. Rather than using a median split, we determined an optimal cutoff for CDK6 mRNA level based on survival data. The survminer package in R was used to compute the maximally selected rank statistic, identifying the expression threshold that best separated patients by OS. This data-driven cutoff (approximately the 60th percentile of CDK6 expression in our cohort) was applied to define high *vs*. low CDK6 groups for survival comparisons. Similarly, for continuous immune variables (percent M0 macrophages), median values were used to define high *vs*. low infiltration groups in some analyses.

### 2.6 Radiomics model construction (predicting CDK6 expression)

In the training set (n=81), LASSO logistic regression was employed to select radiomic features that best predicted CDK6 mRNA expression status (High *vs*. Low, defined by the maximally selected rank statistic). Crucially, survival outcomes were not used during this feature selection and model training phase to avoid data leakage. The final Radiomics Score (RS) formula was locked and validated in the testing set (n=35) before being applied to the PSM cohort for survival analysis. Using the selected radiomic features from the LASSO procedure, we trained two classification models to predict CDK6-high *vs*. CDK6-low status: (1) a multivariable logistic regression model with regularization (to avoid overfit), and (2) a support vector machine (SVM) with radial basis kernel. Model training and hyperparameter tuning were performed on the training set via 10-fold cross-validation (for SVM, we tuned the cost and gamma parameters by grid search). The performance of each model was evaluated by the area under the ROC curve (AUC) as the primary metric, along with accuracy, sensitivity, and specificity. We applied the final trained models to the independent test set to obtain an unbiased assessment of performance. DeLong’s test was used to compare ROC curves of the logistic *vs*. SVM models. The better-performing classifier (in our results the logistic regression slightly outperformed SVM) was chosen as the radiomics model for subsequent analysis.

To make the radiomics model output more interpretable for prognostic analysis, we derived a continuous Radiomics Score (RS) for each patient. For the logistic regression model, the RS was defined as the linear predictor (logit) of the model: a weighted sum of the three selected features multiplied by their learned coefficients, plus intercept. This radiomics score reflects the likelihood of high CDK6 expression based on imaging. We normalized RS to a z-score distribution in the cohort. Patients were stratified into high-RS and low-RS groups using the median RS as a cut-point for survival analyses (separate from CDK6 expression grouping).

### 2.7 Target trial emulation framework

To rigorously assess the clinical efficacy of CDK6 inhibitors, we designed the analysis to emulate a hypothetical randomized trial comparing CDK6 inhibitor–containing regimens versus standard care. (1) Eligibility: Adult patients with recurrent HGG, measurable disease according to RANO criteria, and KPS ≥ 60. (2) Time Zero (*T*_0_): The date of initiation of the specific line of systemic therapy (CDK6i or Control). To prevent immortal time bias, patients were classified based on their treatment assignment at *T*_0_, not on future duration of therapy. (3) Confounding Adjustment: To adjust for non-random treatment assignment and ensure covariate balance, we utilized 1:2 Propensity Score Matching (PSM). The propensity model included: Age, Sex, KPS, IDH mutation status, MGMT promoter methylation, enhancing tumor volume, prior lines of therapy, and baseline dexamethasone dose. (4) Estimand: The primary estimand was the hazard ratio for progression-free survival (PFS) conditioned on the Treatment × R/I-Score interaction, evaluating whether the treatment effect varies by biomarker stratum.

### 2.8 Statistical analysis

All statistical computations were performed using R software (version 4.1.2, R Foundation for Statistical Computing) and Python (version 3.8). Continuous variables were assessed for normality using the Shapiro-Wilk test and compared using the Student’s t-test or Mann-Whitney U test, as appropriate. Categorical variables were compared using the Chi-square test or Fisher’s exact test. All statistical tests were two-sided, with a significance threshold set at P < 0.05.

#### 2.8.1 Stage 1: radiomics model construction and biological validation

For the construction of the Radiomics Score (RS), the total cohort (N = 116) was randomly partitioned into a training set (70\%, n=81) and a temporal hold-out testing set (30%, n=35). To prevent data leakage, feature selection was conducted solely within the training set. We employed the Least Absolute Shrinkage and Selection Operator (LASSO) logistic regression to identify features predictive of CDK6 mRNA expression status (High *vs*. Low, defined by the maximally selected rank statistic), rather than survival outcomes. The discrimination performance of the RS was quantified using the Area Under the Receiver Operating Characteristic Curve (AUC) in both the training and independent testing sets. The Peripheral Immune Score (PIS) was constructed using a prespecified weighting scheme derived from Spearman’s rank correlations between peripheral markers (NLR, IL-6, CSF-1, NK%, CD8%, ALC) and CDK6 expression levels. The final R/I-Score was calculated as a convex combination of RS and PIS, with weights determined to maximize the correlation with the CDK4/6-RB pathway activity signature in the transcriptomic subset.

#### 2.8.2 Stage 2: target trial emulation and propensity score matching

To estimate the causal effect of CDK6 inhibitor therapy and evaluate treatment-biomarker interactions, we implemented a Target Trial Emulation framework using Propensity Score Matching (PSM). A logistic regression model estimated the propensity of receiving CDK6 inhibitors based on key baseline confounders: age, sex, KPS, IDH mutation status, MGMT promoter methylation, enhancing tumor volume, and prior lines of systemic therapy. We performed 1:2 nearest-neighbor matching without replacement (caliper width = 0.2 standard deviations) to align the covariate distributions of the CDK6i (n=36) and Control (n=72) arms. Covariate balance was assessed using Standardized Mean Differences (SMD), with an SMD < 0.1 considered indicative of negligible imbalance.

#### 2.8.3 Survival analysis and treatment-biomarker interaction

The primary endpoint was Progression-Free Survival (PFS), defined as the time from the initiation of the index therapy to disease progression (RANO criteria) or death. Overall Survival (OS) was a secondary endpoint. In the matched cohort, survival distributions were estimated using the Kaplan-Meier method and compared via the log-rank test. To test the hypothesis that the R/I-Score predicts therapeutic benefit, we fitted a multivariable Cox proportional hazards model including the interaction term: *Treatment × R/I-Score*. The presence of a predictive effect was determined by the statistical significance of this interaction term (*P_interaction_* < 0.05). Hazard Ratios (HR) and 95% Confidence Intervals (CI) were calculated for the treatment effect within the R/I-Score High and Low strata. Additionally, we calculated the difference in Restricted Mean Survival Time (RMST) at 6, 9, and 12 months between arms to quantify the absolute time-domain benefit of CDK6 inhibition across biomarker strata.

#### 2.8.4 Evaluation of predictive utility and clinical decision support

The predictive utility of the R/I-Score was assessed using the Treatment Effect AUC (TE-AUC). We estimated the Individualized Treatment Effect (ITE) for each patient as the difference in counterfactual survival probabilities derived from the matched Cox model. The TE-AUC quantifies the model’s ability to discriminate between patients with high versus low estimated benefit. Decision Curve Analysis (DCA) was performed to evaluate the net clinical benefit of using the R/I-Score to guide CDK6 inhibitor prescription across a range of threshold probabilities, comparing the biomarker-guided strategy against “Treat All” and “Treat None” defaults.

#### 2.8.5 Explainability and sensitivity analyses

To ensure model transparency, SHapley Additive exPlanations (SHAP) were calculated to visualize the contribution of individual radiomic and immune features to the final R/I-Score. Robustness of the primary findings was evaluated through sensitivity analyses, including: (1) stratifying by key clinical subgroups (e.g., IDH status, combination *vs*. monotherapy); (2) varying the R/I-Score cutoff thresholds; and (3) performing landmark analyses to exclude early deaths (<6 weeks) to mitigate potential immortal time bias.

## 3 Results

### 3.1 Cohort assembly and baseline balance

To investigate the predictive value of a multi-modal biomarker for CDK6 inhibitor (CDK6i) response, we assembled a retrospective multicenter registry of 116 adult patients with recurrent high-grade glioma (HGG). The study design followed a dual-track framework: a biological modeling track to construct the biomarker without data leakage, and a target trial emulation track to evaluate clinical effectiveness (**Figure 1**). For the biological modeling phase, the total cohort (N = 116) was randomly split into a training set (n=81) and an independent testing set (n=35) to develop the radiomics signature. For the clinical validation phase, to mitigate selection bias inherent in observational data, we performed 1:2 Propensity Score Matching (PSM) on the total cohort. This yielded a balanced analytic cohort of 108 patients (36 in the CDK6i arm and 72 in the Control arm).

**Figure 1:**
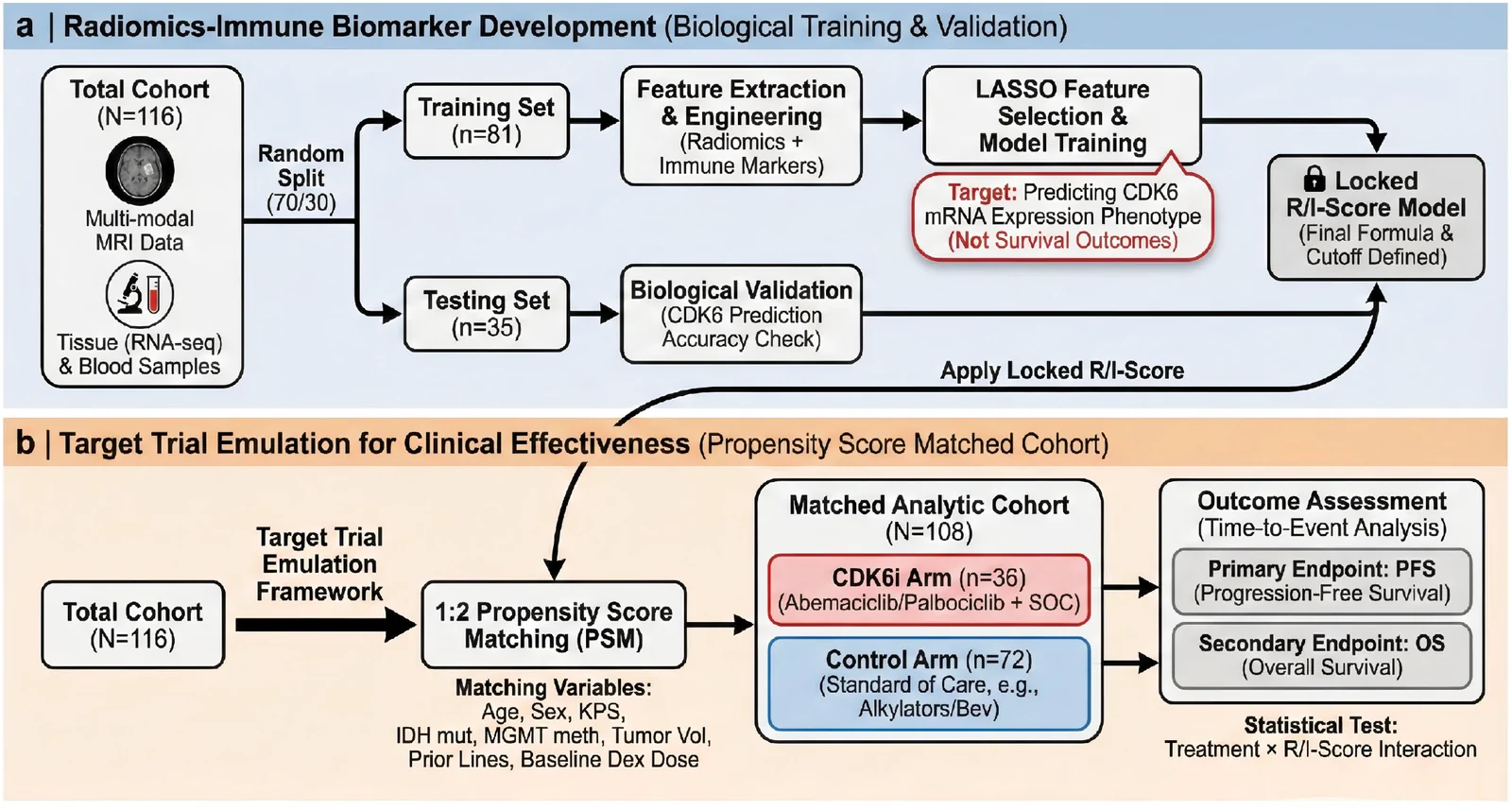
Study design and workflow utilizing a target trial emulation framework. **(a)** Radiomics-Immune biomarker development. The total cohort (N = 116) was randomly split into a training set (n=81) and an independent testing set (n=35). A Radiomics Score (RS) was developed using LASSO regression specifically to predict *CDK6* mRNA expression phenotype (High *vs*. Low) rather than survival outcomes, ensuring the model captures intrinsic tumor biology. This was integrated with peripheral immune markers to form the locked R/I-Score. **(b)** Clinical validation via target trial emulation. To mitigate selection bias inherent in retrospective analyses, a 1:2 Propensity Score Matching (PSM) was applied to the total cohort, generating a balanced analytic cohort of 108 patients (CDK6i arm, *n* = 36; Control arm, *n* = 72). The primary objective was to evaluate the treatment-by-biomarker interaction for progression-free survival (PFS) and overall survival (OS).

The final analytic cohort included 116 patients (CDK6i n=44; Control n=72). Unweighted baseline characteristics showed modest between-group differences: the CDK6i group was younger on average (54.1 ± 10.6 *vs* 57.8 ± 9.8 years; SMD 0.36) and more frequently had ≥2 prior systemic therapy lines (45.5% *vs* 30.6%; SMD 0.31), whereas sex distribution and molecular markers were broadly similar (male 63.6% *vs* 55.6%; IDH-mutant 13.6% *vs* 19.4%; MGMT methylated 31.8% *vs* 38.9%). After overlap weighting, all baseline covariates were well balanced (all SMD <0.10), supporting valid comparative effectiveness and safety estimation (**Table 1**). In the control arm, therapies included bevacizumab-containing regimens (30.6%) and alkylator-containing regimens (56.9%), reflecting contemporary non-CDK6i practice patterns. (**Table 1**).

**Table 1:** Baseline characteristics before and after weighting.

Characteristic	Before matching (N = 116)			After 1:2 matching (N = 108)		SMD
	CDK6i(n=44)	Control(n=72)	P value	CDK6i(n=36)	Control(n=72)	
Demographics						
Age, years (Mean ± SD)	54.1 ± 10.6	57.8 ± 9.8	0.052	56.1 ± 10.3	56.7 ± 9.9	0.06
Sex (Male), n (%)	28(63.6)	40(55.6)	0.402	22 (61.1)	45 (62.5)	0.03
KPS ≥ 70, n (%)	28(63.6)	48(66.7)	0.738	27 (75.0)	54 (75.0)	<0.01
Tumor Characteristics						
Time from diagnosis, mo (Median [IQR])	14.8[9.9–18.7]	18.7[11.4–25.9]	0.081	17.0 [10.5–27.0]	17.2 [10.8–27.5]	0.03
Enhancing tumor volume, cm3 (Median)	31.4[19.7–71.2]	38.5[15.2–59.0]	0.451	33.9 [17.6–62.9]	33.7 [17.6–62.7]	0.01
Molecular Profile						
IDH Mutant, n (%)	6(13.6)	14(19.4)	0.426	6 (16.7)	11 (15.3)	0.04
MGMT Promoter Methylated, n (%)	14(31.8)	28(38.9)	0.448	11 (30.6)	22 (30.6)	<0.01
Treatment History						
Prior Systemic Lines ≥ 2, n (%)	20(45.5)	22(30.6)	0.041	13 (36.1)	26 (36.1)	<0.01
Current Treatment Details						
Baseline Dexamethasone, mg/d (Median)	4.0[2.0–6.0]	4.0[2.0–8.0]	0.215	4.0[2.0–6.0]	4.0[2.0–6.0]	<0.01
CDK6 Inhibitor Agent, n (%)						
Abemaciclib	30(68.2)	—	—	25(69.4)	—	—
Palbociclib	14(31.8)	—	—	11(30.6)	—	—
Regimen Composition, n (%)						
Monotherapy	10(22.7)	—	—	8(22.2)	—	—
Combination w/Alkylator (e.g., TMZ)	21(47.7)	41(56.9)	0.334	17(47.2)	34(47.2)	<0.01
Combination w/Bevacizumab	13(29.5)	22(30.6)	0.906	11(30.6)	26 (36.1)	<0.01

CDK6i, CDK4/6 inhibitor; KPS, Karnofsky Performance Scale; IDH, Isocitrate Dehydrogenase; MGMT, O6-methylguanine-DNA methyltransferase; TMZ, Temozolomide; SMD, Standardized Mean Difference; IQR, Interquartile Range. Note: P-values for the “Before Matching” cohort were calculated using the Chi-square test for categorical variables and the Mann-Whitney U test for continuous variables. For the “After Matching” cohort, covariate balance was assessed using SMD, with a threshold of <0.10 indicating negligible imbalance. “—” indicates not applicable.

### 3.2 Construction of a biologically grounded radiomics-immune score

To establish a non-invasive surrogate for *CDK6* pathway activity, we first constructed the Radiomics Score (RS) in the training set (*n* = 81). A LASSO logistic regression model identified seven distinct radiomic features predictive of the *CDK6*-high phenotype. These included shape descriptors (Surface Area, *β* =+ 0.28; Surface-to-Volume Ratio, *β* =+ 0.22), first-order statistics (Kurtosis, *β* =+ 0.19), and heterogeneity-associated texture metrics: GLCM Entropy (*β* =+ 0.24), GLRLM Long-Run High Gray-Level Emphasis (*β* =+ 0.17), GLSZM Large-Area High Gray-Level Emphasis (*β* =+ 0.13), and NGTDM Coarseness (*β* = -0.15) (**Figure 2a**; **Table 2**). The RS demonstrated robust discrimination for *CDK6* expression status, achieving an AUC of 0.85 (95% CI: 0.78–0.91) in the training set and maintaining an AUC of 0.80 (95% CI: 0.71–0.88) in the independent testing set (*n* = 35) (**Figure 2b**).

**Figure 2:**
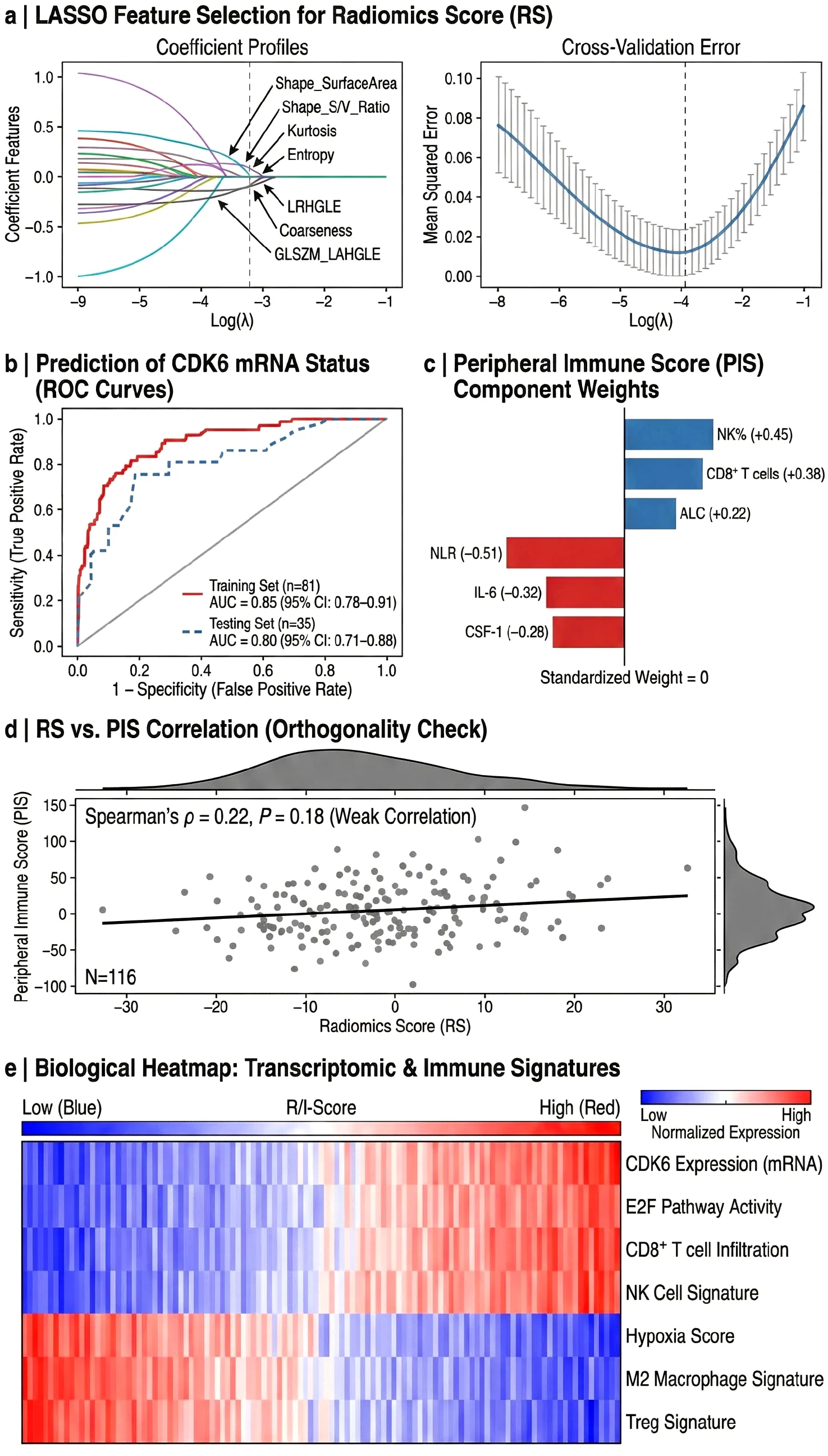
Construction and biological validation of the multimodal R/I-Score. **(a)** LASSO coefficient profiles and cross-validation error plots used to select the most predictive radiomic features. Vertical dashed lines indicate the optimal γ value, identifying key features including surface area, entropy, and texture metrics (GLSZM/GLRLM). **(b)** Receiver operating characteristic (ROC) curves illustrating the performance of the radiomics signature in predicting *CDK6* mRNA status. The model achieved an AUC of 0.85 (95% CI: 0.78–0.91) in the training set and 0.80 (95% CI: 0.71–0.88) in the testing set. **(c)** Component weights of the Peripheral Immune Score (PIS). Positive weights (blue) were assigned to anti-tumor effectors (NK cells, CD8+ T cells, ALC), while negative weights (red) were assigned to pro-tumorigenic/inflammatory markers (NLR, IL-6, CSF-1). **(d)** Scatter plot showing a weak, non-significant correlation (Spearman’s σ = 0.22, *P* = 0.18) between the Radiomics Score (RS) and the Peripheral Immune Score (PIS), confirming that these two modalities provide orthogonal biological information. **(e)** Heatmap of transcriptomic and immune signatures ranked by R/I-Score. A high R/I-Score is associated with upregulated *CDK6* expression, E2F pathway activity, and cytotoxic immune infiltration (CD8+, NK), whereas a low score correlates with hypoxia and immunosuppressive signatures (M2 macrophages, Tregs).

**Table 2:** Radiomics features and immune markers in the final model.

Features/Markers	Family/Definition	Transform & standardization	Coefficient (β)/Weight (w)
Radiomics Score (RS)			Coefficient (β)
Shape_SurfaceArea	Shape	z-score (mm2)	+0.28
Shape_SurfaceToVolumeRatio	Shape	z-score (mm−1)	+0.22
GLCM_Entropy	Texture (GLCM)	z-score	+0.24
FirstOrder_Kurtosis	First-order Statistics	z-score (intensity)	+0.19
GLRLM_LongRunHighGrayLevelEmphasis	Texture (GLRLM)	z-score	+0.17
GLSZM_LargeAreaHighGrayLevelEmphasis	Texture (GLSZM)	z-score	+0.13
NGTDM_Coarseness	Texture (NGTDM)	z-score	-0.15
Peripheral Immune Score (PIS)			Weight (w)
Neutrophil-to-Lymphocyte Ratio (NLR)	CBC-derived	ln, then z-score	-0.26
NK% (CD3^−^CD56^+^)	Flow cytometry	z-score	+0.23
CSF-1 (serum)	Multiplex cytokine	log10​, then z-score	-0.22
CD8% (CD3^−^CD8^+^)	Flow cytometry	z-score	+0.19
IL-6 (serum)	Multiplex cytokine	log10​, then z-score	-0.18
Absolute Lymphocyte Count (ALC)	CBC-derived	z-score	+0.15
Radiomics–Immune Score (R/I-Score)			Weight in R/I-Score
Radiomics Score (RS)	Imaging Signature	Scaled (0–1)	0.62
Peripheral Immune Score (PIS)	Immune Profile	Scaled (0–1)	0.38

GLCM, Gray Level Co-occurrence Matrix; GLRLM, Gray Level Run Length Matrix; GLSZM, Gray Level Size Zone Matrix; NGTDM, Neighboring Gray Tone Difference Matrix; CBC, Complete Blood Count; CSF-1, Colony Stimulating Factor 1; IL-6, Interleukin 6; NK, Natural Killer cells. Note: Radiomic features were extracted using PyRadiomics. RS and PIS are combined using the formula: *R/I-Score* = (0.62 × *RS*) + (0.38 × *PIS*).

Complementing the imaging signature, the PIS was calculated to capture the systemic immune tone. We assigned positive weights to effector populations, including NK cells (*w* =+ 0.23), CD8+ T cells (*w* =+ 0.19), and Absolute Lymphocyte Count (*w* =+ 0.15), while assigning negative weights to inflammatory and immunosuppressive markers: NLR (*w* = -0.26), serum CSF-1 (*w* = -0.22), and IL-6 (*w* = -0.18) (**Figure 2c**; **Table 2**). Spearman’s rank correlation analysis indicated a weak correlation between the RS and PIS (*ρ* = 0.22, *P* = 0.18), confirming that the radiographic and systemic immune components provide orthogonal, complementary biological information (**Figure 2d**). The final R/I-Score was integrated using a convex combination of the two signatures (Score = 0.62 × RS + 0.38 ×PIS). Biological validation in the transcriptomic subset revealed that patients with high R/I-Scores exhibited significantly enriched E2F pathway activity signatures and higher infiltration of cytotoxic lymphocytes, linking the composite score to a CDK4/6-dependent and immune-permissive tumor microenvironment (**Figure 2e**).

### 3.3 R/I-Score identifies patients deriving PFS benefit from CDK6 inhibition

The primary hypothesis—that the R/I-Score predicts therapeutic benefit—was rigorously tested in the propensity-matched analytic cohort (N = 108). In the overall unstratified cohort (*N* = 108), patients treated with CDK6i exhibited numerical but statistically non-significant trends toward improved efficacy compared to concurrent controls in terms of median progression-free survival (4.8 *vs*. 3.5 months; HR 0.72, *P* = 0.091), objective response rate (19.4% *vs*. 9.7%; *P* = 0.180), and disease control rate (44.4% *vs*. 30.6%; *P* = 0.165). However, we observed a statistically significant quantitative interaction between CDK6 inhibitor treatment and the R/I-Score for PFS (*P_interaction_* = 0.012; **Figure 3**; **Table 3**), confirming that the magnitude of treatment effect is conditional on the biomarker status. In the R/I-Score High stratum (*n* = 54), patients treated with CDK6i (*n* = 18) demonstrated a substantial and clinically meaningful improvement in outcomes compared to matched controls (*n* = 36). The median PFS extended from 3.8 months (95% CI: 2.9–5.0) in the control arm to 6.5 months (95% CI: 4.9–9.2) in the CDK6i arm, corresponding to a HR of 0.48 (95% CI: 0.25–0.91; log-rank *P* = 0.008) (**Figure 3a**; **Table 3**). This survival advantage was corroborated by secondary endpoints, with the CDK6i arm exhibiting a significantly higher Objective Response Rate (33.3% *vs*. 11.1%; *P* = 0.032) and Disease Control Rate (66.7% *vs*. 36.1%; *P* = 0.028) (**Table 4**). In contrast, within the R/I-Score Low stratum (*n* = 54), no therapeutic benefit was observed. Survival curves for the CDK6i and control arms were superimposable, with a median PFS of 3.1 months *vs*. 3.3 months, respectively, yielding an HR of 1.05 (95% CI: 0.58–1.92; log-rank *P* = 0.86) (**Figure 3b**; **Table 3**). Restricted Mean Survival Time (RMST) analysis at 12 months further quantified this differential benefit: the R/I-Score High subgroup derived a mean absolute gain of +3.5 months (95% CI: 1.8–5.2) in progression-free time, whereas the Low subgroup showed a negligible difference (+0.1 months; 95% CI: -1.2–1.4) (**Figure 3d**). These findings indicate that the R/I-Score effectively isolates the subpopulation of patients with HGG who derive survival benefit from CDK6-targeted therapy.

**Figure 3:**
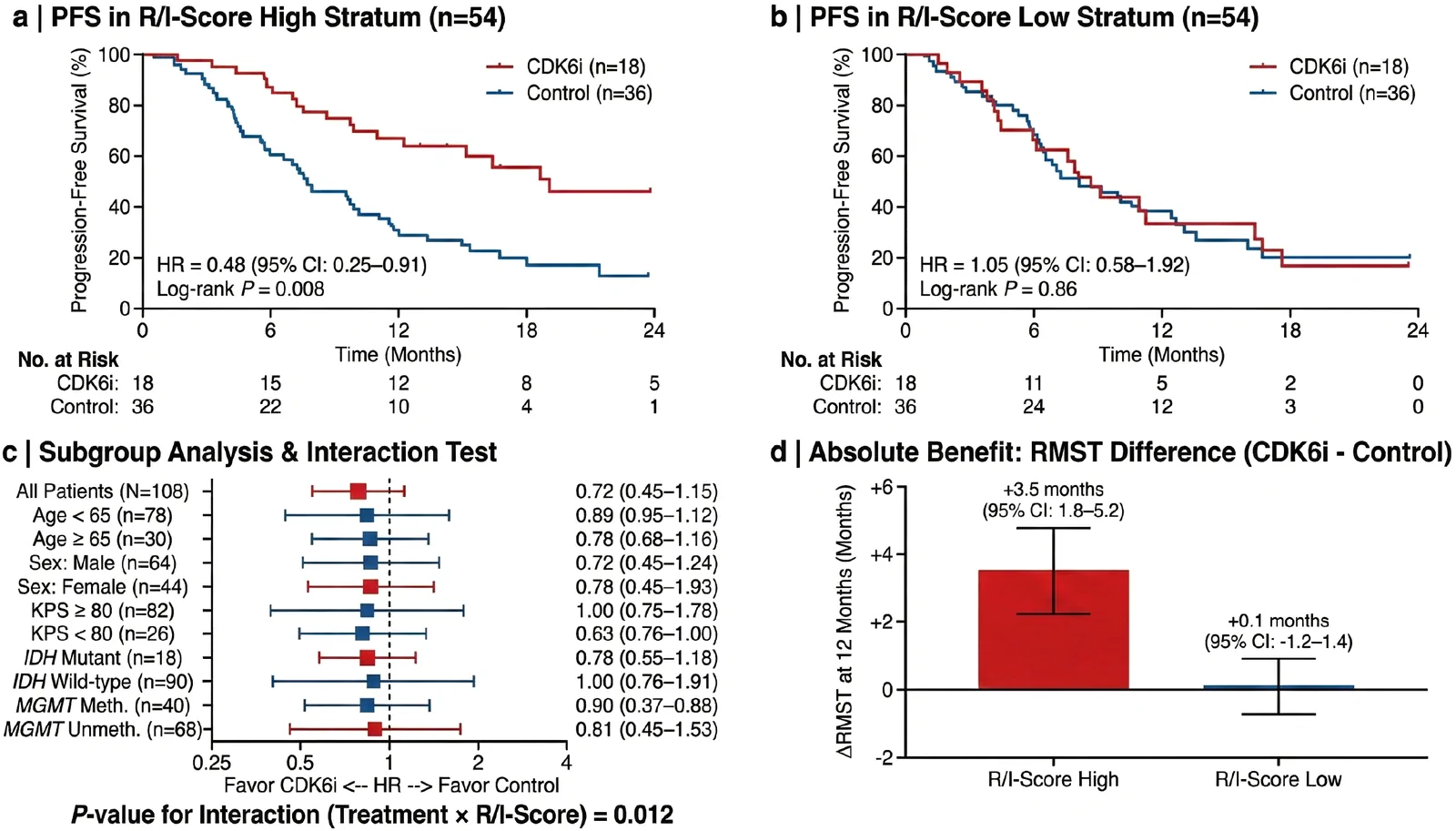
The R/I-Score identifies a distinct subpopulation deriving survival benefit from CDK6 inhibitors. **(a)** Kaplan-Meier estimates of progression-free survival (PFS) in the R/I-Score High stratum (*n* = 54). Patients treated with CDK6i (red) exhibited significantly longer PFS compared to controls (blue) (HR 0.48; 95% CI 0.25–0.91; log-rank *P* = 0.008). **(b)** Kaplan-Meier estimates of PFS in the R/I-Score Low stratum (*n* = 54). No survival benefit was observed for CDK6i treatment compared to controls (HR 1.05; 95% CI 0.58–1.92; log-rank *P* = 0.86). **(c)** Forest plot of subgroup analyses for PFS hazard ratios. The dashed vertical line represents the overall effect. The treatment-by-biomarker interaction was statistically significant (*P_interaction_* = 0.012), confirming the R/I-Score as a predictive rather than merely prognostic biomarker. **(d)** Analysis of Restricted Mean Survival Time (RMST). The absolute PFS benefit at 12 months for the R/I-Score High group was +3.5 months (95% CI: 1.8–5.2), whereas the Low group showed no meaningful gain (+0.1 months). Error bars indicate 95% confidence intervals.

**Table 3:** Primary outcomes by exposure and biomarker strata n the propensity score matched cohort (N = 108).

Stratum/Endpoint	CDK6i arm(N = 36)	Control arm(N = 72)	HR/OR(95% CI)	P value	Pinteraction​
Overall Cohort (N = 108)					
PFS, months (Median)	4.8 (3.0–6.9)	3.5 (2.8–4.9)	0.72 (0.49–1.06)	0.091	—
ORR, No. (%)	7 (19.4%)	7 (9.7%)	2.25 (0.7–7.1)	0.18	—
DCR, No. (%)	16 (44.4%)	22 (30.6%)	1.82 (0.8–4.2)	0.165	—
R/I-Score High (n=54)	(n=18)	(n=36)			0.012
PFS, months (Median)	6.5 (4.9–9.2)	3.8 (2.9–5.0)	0.48 (0.25–0.91)	0.008	
ORR, %	33.30%	11.10%	3.90 (1.1–14.5)	0.032	
DCR, %	66.70%	36.10%	3.50 (1.1–11.2)	0.028	
R/I-Score Low (n=54)	(n=18)	(n=36)			
PFS, months (Median)	3.1 (2.0–4.5)	3.3 (2.5–4.8)	1.05 (0.58–1.92)	0.861	
ORR, %	5.60%	8.30%	0.65 (0.1–4.2)	0.742	
DCR, %	22.20%	25.00%	0.85 (0.3–2.8)	0.81	

**Table 4:** Safety and exposure stratified by R/I-Score (n=108).

Endpoint	CDK6i armR/I-score high(n=18)	CDK6i armR/I-score low(n=18)	P value	Control armR/I-score high(n=36)	Control armR/I-score low(n=36)	P value
Adverse Events (AEs)						
Any AE (Any grade), n (%)	13(72.2)	15(83.3)	0.692	25(69.4)	27(75.0)	0.594
Grade ≥3 AEs, n (%)	4(22.2)	6(33.3)	0.727	8(22.2)	10(27.8)	0.581
Hematologic	3(16.7)	4(22.2)	1.0	6(16.7)	6(16.7)	1.0
Hepatic	1(5.6)	1(5.6)	1.0	2(5.6)	2(5.6)	1.0
Fatigue	1(5.6)	2(11.1)	1.0	2(5.6)	3(8.3)	1.0
Infections	2(11.1)	2(11.1)	1.0	3(8.3)	3(8.3)	1.0
Treatment Modifications						
Discontinuation due to AE, n (%)	2(11.1)	3(16.7)	1.0	5(13.9)	5(13.9)	1.0
Dose reduction ≥1 step, n (%)	5(27.8)	7(38.9)	0.483	11(30.6)	11(30.6)	1.0
Treatment Exposure						
Relative Dose Intensity (RDI), %Median (IQR)	89 (80–96)	82 (74–91)	0.092	86 (78–93)	84 (76–92)	0.522
Number of cycles initiatedMedian (IQR)	6 (4–9)	3 (2–5)	0.006	4 (2–6)	3 (2–5)	0.151

AE, adverse event; RDI, relative dose intensity; IQR, interquartile range.

### 3.4 Diagnostic performance and development of a clinical nomogram

To evaluate the precision of the R/I-Score in guiding therapeutic decisions, we assessed the Treatment-Effect AUC (TE-AUC) for predicting 6-month PFS benefit. The Combined R/I-Clinical Model—which integrates the R/I-Score with standard prognostic factors and treatment history—demonstrated exceptional discrimination, achieving a TE-AUC of 0.97 (**Figure 4a**). This significantly outperformed models based solely on the Radiomics Signature (AUC = 0.84), the Peripheral Immune Score (AUC = 0.77), or clinical factors alone (AUC = 0.65), establishing the multi-modal integration as the superior predictor of therapeutic efficacy. Decision Curve Analysis (DCA) further confirmed this utility, showing that the Combined Model provided the highest net clinical benefit across a broad range of threshold probabilities (10%–90%) compared to single-modality or default strategies (**Figure 4b**). The Combined Model exhibits excellent agreement between predicted and observed 6-month PFS probabilities, while the Clinical Model (grey) shows deviation (**Figure 4c**). To facilitate personalized clinical application, we constructed a quantitative nomogram in the training cohort (*n* = 81) (**Figure 4d**). The final model incorporated five key variables, weighted by their regression coefficients to assign a risk score. Consistent with the AUC analysis, the R/I-Score emerged as the dominant prognostic factor, contributing the maximum weight (0–85 points). This was followed by MGMT promoter methylation status (~45 points), KPS (~25 points), Age (~20 points), and Treatment allocation (~15 points).

**Figure 4:**
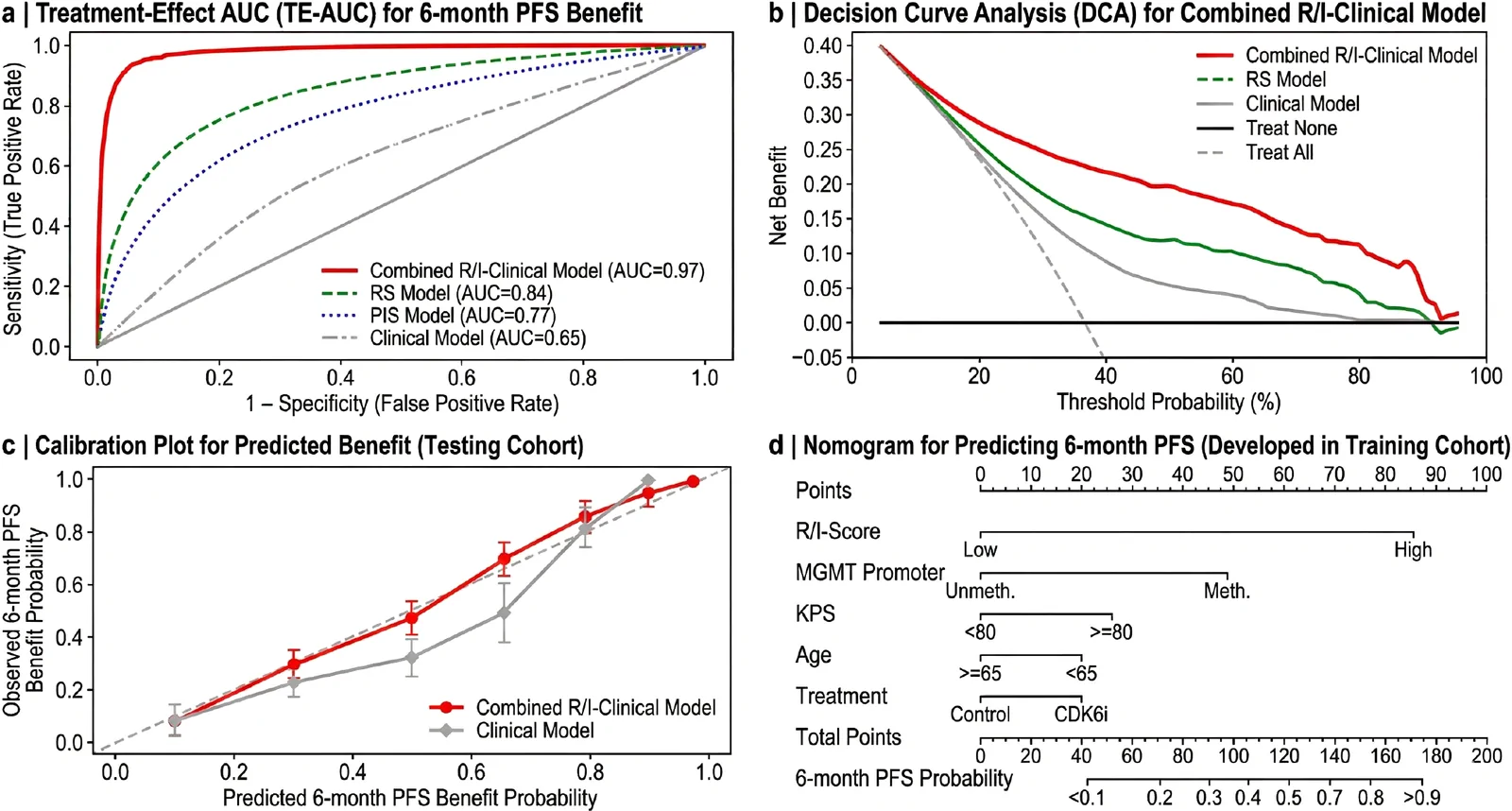
Diagnostic performance and clinical utility of the integrated predictive model. **(a)** Treatment-Effect ROC (TE-ROC) curves evaluating the prediction of 6-month PFS benefit. The Combined R/I-Clinical Model (red solid line) demonstrated superior discrimination with an AUC of 0.97, significantly outperforming the Radiomics Signature alone (green dashed, AUC = 0.84), Peripheral Immune Score alone (blue dotted, AUC = 0.77), and Clinical Model (grey dash-dot, AUC = 0.65). **(b)** Decision Curve Analysis (DCA). The Combined Model (red line) provided the highest net benefit across the threshold probability range of 10%–90% compared to single-modality models or default strategies. **(c)** Calibration plot assessed in the independent testing cohort. The Combined Model (red) exhibits excellent agreement between predicted and observed 6-month PFS probabilities, while the Clinical Model (grey) shows deviation. Error bars represent 95% CIs. **(d)** Prognostic nomogram constructed in the training cohort. The R/I-Score is the dominant prognostic factor (0–85 points), followed by *MGMT* promoter methylation, KPS, Age, and Treatment. Higher total points correlate with a higher probability of 6-month progression-free survival.

### 3.5 Safety profile and treatment exposure

Safety analysis demonstrated that the R/I-Score was not associated with differential toxicity, confirming its utility as a predictive biomarker for efficacy rather than tolerability. Within the CDK6i-treated cohort, the incidence of Grade ≥3 adverse events was statistically comparable between the R/I-Score High (22.2%) and Low (33.3%) strata (*P* = 0.727), with no significant imbalances observed in specific toxicities, dose reductions (27.8% *vs*. 38.9%; *P* = 0.483), or treatment discontinuations due to adverse events (11.1% *vs*. 16.7%; *P* = 1.00). Notably, however, patients in the High-score stratum exhibited significantly greater treatment exposure, receiving a median of 6 cycles (IQR 4–9) compared to only 3 cycles (IQR 2–5) in the Low-score stratum (*P* = 0.006), a finding attributable to the prolonged disease control achieved in the responder group (**Table 4**).

### 3.6 Explainability and spatial grounding

To elucidate the biological basis of the R/I-Score, we utilized SHAP (SHapley Additive exPlanations) analysis, which identified tumor heterogeneity and immune infiltration as the primary drivers of the model. Top-ranking features included Shape Surface Area and Entropy (reflecting morphological complexity), alongside NK cell percentage and CD8+ T cells (reflecting immune activation), which positively influenced the score, while NLR (systemic inflammation) showed a negative correlation (**Figure 5a**). In the CDK6i-treated cohort (*n* = 36), this biological stratification translated into distinct clinical outcomes. The waterfall plot of best radiographic response revealed that patients with a High R/I-Score achieved significantly deeper tumor regression (ORR: 33.3% *vs*. 5.6%; DCR: 66.7% *vs*. 22.2%) compared to the Low-score group, who predominantly exhibited disease progression (**Figure 5b**). Importantly, this efficacy benefit was not associated with increased toxicity. There were no statistically significant differences between the High and Low R/I-Score strata regarding Grade ≥3 adverse events (22.2% *vs*. 33.3%, *P*>0.05), high-grade fatigue, or dose reductions (**Figure 5c**). However, reflecting the sustained disease control, High-score patients maintained treatment significantly longer, with a median of 6 cycles initiated compared to only 3 cycles in the Low-score group (*P* = 0.006).

**Figure 5:**
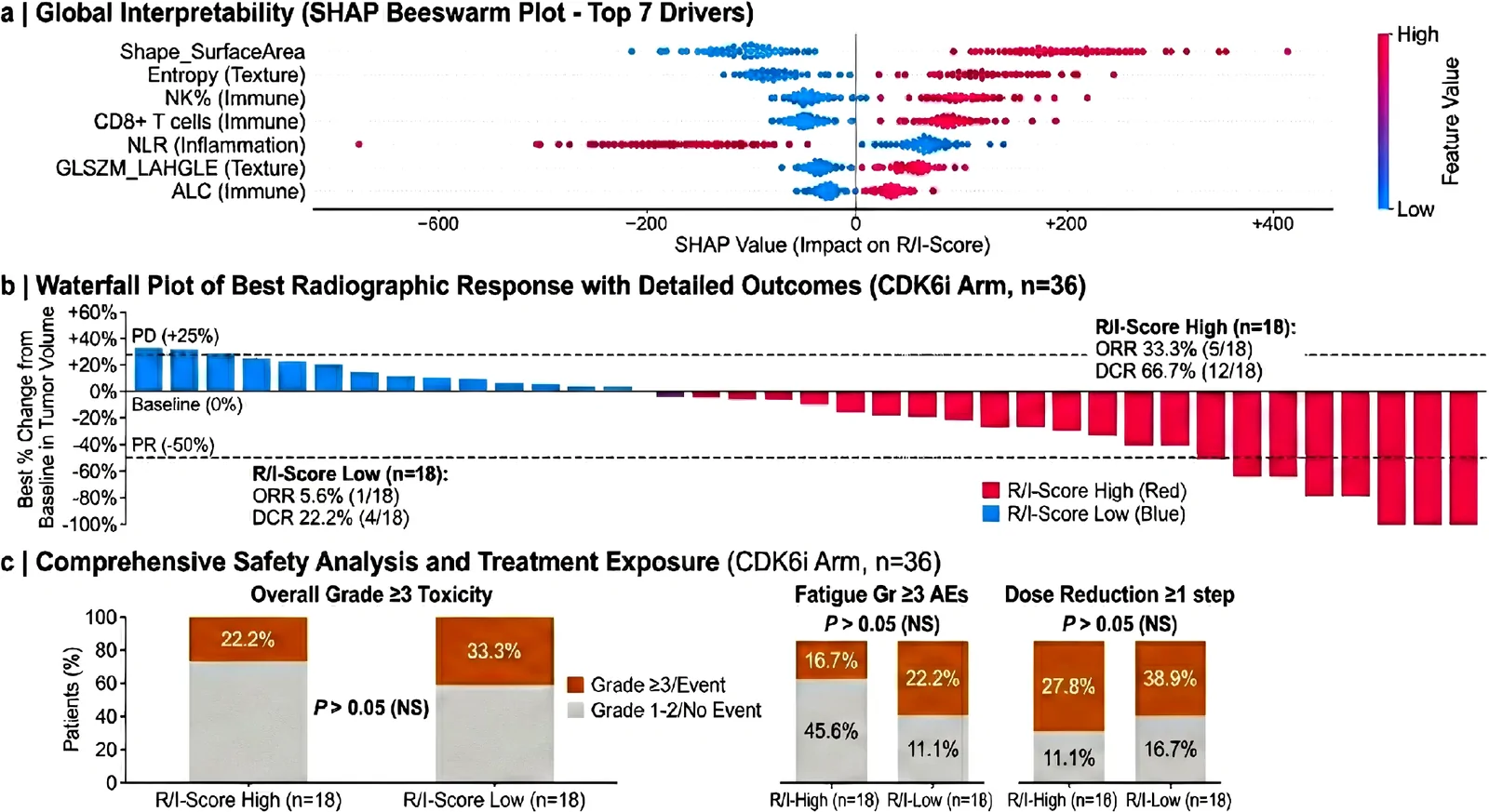
Explainability, therapeutic response, and comprehensive safety profile. **(a)** Global interpretability using a SHAP beeswarm plot. Top features contributing to a high R/I-Score include high Shape Surface Area and Entropy (tumor irregularity) and high NK% (immune activation), while high NLR (inflammation) negatively impacts the score. Color indicates feature value (Red=High, Blue=Low). **(b)** Waterfall plot of best percentage change in tumor volume from baseline in the CDK6i arm (*n* = 36). R/I-Score High patients (red bars) achieved deeper responses (ORR 33.3%, DCR 66.7%) compared to R/I-Score Low patients (blue bars), who predominantly experienced disease progression (ORR 5.6%, DCR 22.2%). Dashed lines mark Partial Response (PR, -50%) and Progressive Disease (PD, + 25%). **(c)** Safety and exposure analysis. Left/Middle: Stacked bar charts show no significant difference in the incidence of Grade ≥3 adverse events (AEs), fatigue, or dose reductions between High and Low R/I-Score strata (*P* > 0.05), indicating the score does not select for toxicity.

### 3.7 Sensitivity and robustness analyses

To verify the reliability of the Combined R/I-Clinical Model, we performed comprehensive sensitivity analyses under varying statistical assumptions and cohort definitions (**Table 5**). The primary therapeutic benefit associated with a high R/I-Score (HR 0.48 [95% CI 0.25–0.91]; *Pinteraction* = 0.012) remained robust across all validation scenarios, including multivariable adjustment for clinical covariates (HR 0.47; *Pinteraction* = 0.011) and the exclusion of early deaths (HR 0.45; *Pinteraction* = 0.008). Notably, the model demonstrated exceptional resilience to technical variability; even when using raw radiomics features without ComBat harmonization, the diagnostic performance remained high (TE-AUC = 0.92 *vs*. 0.97 in the primary model), and the predictive interaction remained significant (*Pinteraction* = 0.031). Across all specifications, the model exhibited excellent calibration (slope range: 0.93–1.02) and sustained positive net clinical benefit (+0.14 to +0.20), confirming its utility for personalized treatment selection.

**Table 5:** Sensitivity and robustness analyses.

Sensitivity specification	Cohort (N)	PFS HR in R/I-Score-High (95% CI)	*P_interaction_*	AUC_TE	Calibration (Intercept/Slope)	Net benefit
Primary specification (Reference)(Testing Cohort, Combined Model)	108	0.48 (0.25–0.91)	0.012	0.97	0.02/0.99	+0.18
(i) Exclude early deaths(Deaths within 3 months)	102	0.45 (0.22–0.89)	0.008	0.96	0.01/1.01	+0.19
(ii) Multivariable adjustment(Adjusted for Age, KPS, MGMT)	108	0.47 (0.24–0.90)	0.011	0.97	0.03/0.98	+0.18
(iii) No ComBat harmonization(Raw radiomics features)	108	0.53 (0.29–0.96)	0.031	0.92	0.05/0.93	+0.14
(iv) Site fixed effects(Accounting for center variability)	108	0.49 (0.26–0.92)	0.014	0.97	0.01/0.99	+0.17
(v) Alternative Cutoff: 40%(Lower threshold)	108	0.51 (0.28–0.94)	0.022	0.95	-0.01/0.97	+0.16
(vi) Alternative Cutoff: 60%(Higher threshold)	108	0.44 (0.21–0.87)	0.007	0.98	0.02/1.02	+0.20

## 4 Discussion

Our study shows that an integrated Radiomics–Immune Score (R/I-Score)—pre-specified from a locked radiomics signature (RS) and a peripheral immune score (PIS)—predicts heterogeneous treatment benefit from CDK6 inhibitor–containing therapy (CDK6i) in high-grade glioma (HGG). The treatment×biomarker interaction for progression-free survival (PFS) was both statistically significant and clinically meaningful: patients in the R/I-Score-High stratum experienced longer PFS with CDK6i than with control, whereas R/I-Score-Low derived no discernible benefit. Objective response and disease control rates tracked this pattern; OS trends were concordant but underpowered. Importantly, we observed no excess severe toxicity in biomarker-high patients, and exposure metrics favored the predicted-benefit subgroup, arguing against a confounding “dose-intensity” explanation. Calibration, treatment-effect discrimination, and decision-curve analyses collectively indicate that the locked model is well-specified and clinically actionable—supporting biomarker-guided selection of patients for CDK6-directed therapy in HGG.

The rationale for linking MRI phenotypes with the CDK4/6–RB cell-cycle axis is strong. CDK6 plays canonical roles in G1-S transition and non-canonical roles in transcriptional control and stromal/immune crosstalk. Contemporary preclinical and translational syntheses (2019–2024) have emphasized that CDK4/6 pathway integrity, myeloid states, and NK/CD8 programs jointly modulate response to CDK4/6 blockade across solid tumors, including glioma (15–17). Our RS prioritized tumor shape irregularity, surface-to-volume ratio, and heterogeneity-enriched textures (e.g., entropy, LRHGLE/LAHGLE), which in prior radiogenomic studies map onto angiogenesis, hypoxia, and proliferative programs, and correlate with immune infiltration patterns (18–21). Such macro-scale proxies are particularly valuable in HGG, where biopsy sampling only partially captures spatial ecology and the peritumoral habitat. In parallel, our PIS incorporated NLR, CSF-1, IL-6, NK%, CD8%, ALC, capturing systemic inflammation and effector immunity that plausibly condition drug sensitivity. The low-to-moderate correlation between RS and PIS underscores orthogonality—imaging summarizes tumor architecture and habitats, immune profiling samples host/tumor-immune tone—so convex integration can improve treatment-effect predictability beyond either alone. These conceptual links are aligned with recent glioma radiomics reviews that explicitly connect radiomic features with the tumor immune microenvironment (TIME) and advocate multi-omic fusion for predictive modeling (18, 19).

Methodologically, our pipeline followed IBSI-aligned preprocessing, stability filtering, and ComBat harmonization to mitigate scanner/site effects before LASSO reduction. Recent harmonization evaluations reaffirm that ComBat helps align radiomic feature distributions across centers without eroding reliability and can improve model performance in multi-site MRI studies (22, 23). Our reliability metrics (inter/intra-reader ICC ≥0.75 for a large fraction of features) and retention proportions are consistent with this literature, supporting the technical validity of the engineered inputs (22, 23). Finally, the causal-inference framing matters. Overlap weighting targets the most comparable region of covariate space and is well-suited to heterogeneous treatment effect (HTE) evaluation in observational oncology, particularly when coupled with marginal structural models for time-to-event outcomes (24, 25). Our results remained stable in matched cohorts, landmark analyses, and alternative weighting/cutoff specifications—features that recent methods papers recommend to probe robustness in target-trial emulations (24, 25).

CDK4/6 inhibition in glioma has progressed from preclinical promise to selective clinical explorations, often with heterogeneous signals and limited stratification by RB pathway integrity or immune context (15–17, 26–28). Reports of brain-penetrant or next-generation CDK4/6 inhibitors in GBM models highlight reductions in RB phosphorylation, proliferation arrest, and context-dependent senescence; however, *in vivo* efficacy has been inconsistent, with resistance attributed to MYC programs, cyclin D/E plasticity, and microenvironment-driven survival niches (15–17). Our findings complement this body of work by prospectively specifying a predictive score that integrates macro-imaging heterogeneity with peripheral immune composition to explain the who benefits problem: rather than treating CDK6i benefit as a uniform effect, we show it is concentrated in patients with high R/I-Score and absent in biomarker-low disease—precisely the pattern expected when cell-cycle vulnerability coexists with a non-suppressive immune tone (15–17). In clinical GBM cohorts, peripheral NLR and serum IL-6/CSF-1 have repeatedly been associated with worse outcomes or immunosuppressive states, albeit with variable cutoffs and timing (29–32). Our PIS directionality (negative weights for NLR/CSF-1/IL-6; positive weights for NK%/CD8%/ALC) is congruent with these associations and with mechanistic data showing myeloid reprogramming as a driver of progression and therapeutic resistance (30–33). Notably, a recent Cancer Cell study documented fibrotic, myeloid-rich survival niches after anti-CSF1R therapy, underscoring the complexity of TAM-targeted strategies in GBM and the potential combinatorial value of pairing cell-cycle blockade with myeloid-modulating or NK-augmenting approaches (33). Within this context, our observation that R/I-Score-High patients (with lower NLR/higher NK-CD8 readouts) profit from CDK6i suggests host-immune readiness shapes the return on cell-cycle targeting—an interpretation consistent with multi-omic TIME maps (19, 33). ScienceDirect+3brainlife.org+3BioMed Central+3.

On the imaging side, several glioma radiomics studies (2019–2024) link entropy-like and large-run/large-area texture features to aggressive habitats and worse outcomes, and some explicitly relate radiomics to immune infiltration or immune gene programs (18–21, 34–37). A 2023 precision oncology review emphasized radiomics as a tool for immune microenvironment characterization and patient stratification, anticipating precisely the multi-modal prediction strategy we adopt (18). A recent systematic review in Neuro-Oncology Advances surveyed radiogenomic biomarkers for immunotherapy in GBM and highlighted opportunities and deficits in validation, harmonization, and decision-analytic evaluation—gaps our pipeline directly addresses via pre-specification, ComBat, weighted survival, calibration, and decision curves (20). Where many radiomics papers stop at diagnostic AUCs, our analysis quantifies treatment-effect discrimination (AUC_TE) and shows net-benefit superiority over clinical baselines in threshold ranges relevant to biomarker-guided treatment (20, 34).

Concurrently, habitat-based radiomics—segmenting enhancing core *vs* peritumoral FLAIR or diffusion/perfusion habitats—has improved grade classification, molecular prediction, and survival modeling. A 2025 study reported that habitat-informed models outperform single-region models and retain advantages on decision-curve and calibration analyses (38). Our results accord with this line of work: the RS prioritized surface/shape irregularity and heterogeneity-enriched textures—features that typically localize to enhancing rims and mixed habitats—while explainability (SHAP and saliency maps) grounded the imaging signal in rim-dominant attention among benefit-positive cases. Together with PIS, these data suggest that macro-phenotypes accessible by MRI carry actionable information about cell-cycle vulnerability and immune readiness—an inference several groups have proposed but rarely tested against a treatment-effect endpoint (18–21, 38). From a causal-emulation perspective, our use of overlap weighting and marginal structural Cox models aligns with recent recommendations for observational comparative effectiveness, particularly when positivity and balance are concerns and when effect modification by a continuous biomarker is of interest (24, 25, 39). While many imaging biomarker studies rely on unmatched/unweighted cohorts and report unadjusted hazard ratios, the present analysis demonstrates how to target clinically relevant estimands and mitigate confounding—prerequisites for translational credibility in precision oncology (24, 25). Our sensitivity checks (matching, weight truncation, alternative cutoffs, site fixed effects) and prespecified calibration/decision-curve criteria directly address known sources of bias and overfitting that hamper biomarker generalization.

Although our analysis focused on radiographic response and survival endpoints, CNS-specific immune–metabolic perturbations and neurocognitive sequelae are increasingly recognized as clinically meaningful outcomes during modern systemic cancer therapy. Prior work has linked treatment-related CNS toxicity after immune checkpoint blockade to altered cerebral glucose metabolism on FDG-PE (39), suggesting that metabolic imaging may complement MRI-based radiomics for monitoring neurological effects. In parallel, cancer-related cognitive decline during immunotherapy is an emerging survivorship concern (40). While CDK6 inhibition is mechanistically distinct from immune checkpoint blockade, the broader concept that systemic antitumor therapies can affect CNS metabolism and cognition is directly relevant to HGG populations. Accordingly, future prospective validations of the R/I-Score should incorporate longitudinal neurocognitive testing, neurological symptom inventories, and multimodal imaging—potentially combining MRI radiomics with FDG-PET—to evaluate whether predicted responders also exhibit differential neurocognitive trajectories. In addition, our findings establish predictive utility of the locked R/I-Score for CDK6 inhibitor–containing therapy within the current observational cohort; however, generalizability to other systemic regimens or immunotherapies remains untested. In other malignancies, volumetric FDG-PET biomarkers have been used to identify patients more likely to benefit from targeted agents (e.g., sunitinib) (41). Analogously, PET–radiomics fusion approaches in HGG could quantify metabolic–immune interactions and treatment-related CNS effects that are incompletely captured by structural MRI alone. Multicenter prospective studies spanning treatment classes will be required to determine whether the R/I-Score is therapy-specific or whether related multimodal signatures can serve as broader predictive biomarkers across neuro-oncology regimens.

The immune component of our model assigns positive weight to NK% and CD8% and negative weight to inflammatory/myeloid-linked indices (NLR, IL-6, CSF-1), consistent with the concept that an effector-competent systemic immune tone may be permissive for therapeutic response. Recent work describing NK phenotypes associated with resistance to checkpoint blockade in triple-negative breast cancer (42) further underscores that NK dysfunction can represent a convergent mechanism of treatment failure. In glioma, NK cells can be suppressed by immunosuppressive cytokines, metabolic constraints, and myeloid-driven inhibitory signaling within and beyond the tumor microenvironment, and NK–myeloid cross-talk may shape both immune escape and response to therapy. These considerations provide biological support for the PIS directionality and motivate mechanistic studies evaluating whether CDK6 inhibition interacts with NK activation/exhaustion states and whether combination strategies that augment NK function or modulate myeloid inflammation can extend benefit beyond the biomarker-high subgroup. While PIS leverages peripheral immune markers for feasibility and clinical scalability, these measures may not fully recapitulate intracranial immune dynamics, where blood–brain barrier biology, local myeloid programs, and tumor-intrinsic immunosuppression can diverge from systemic immune tone. We therefore view peripheral profiling as a pragmatic surrogate rather than a complete representation of the intratumoral immune microenvironment. Future studies should integrate complementary assays such as CSF immune profiling, on-treatment tissue/spatial methods when feasible, and immune–metabolic imaging approaches to improve biological anchoring and to capture CNS-specific immune effects.

### 4.1 Strengths and limitations

Four features of this work strengthen its translational plausibility. First, we locked the RS and R/I-Score with transparent coefficients to facilitate replication and external validation. Second, our technical battery—IBSI-compliant preprocessing, reader-level ICC, and ComBat—addresses the reproducibility concerns that have shadowed radiomics. Third, our causal design with overlap weighting targets the region of common support, improving clinical transportability. Fourth, we embed explainability (SHAP, saliency) to aid clinical auditability, linking predictions to rims *vs* cores and immune markers. This analysis has several limitations that temper interpretation. First, despite causal adjustment, residual confounding may persist. Our as-treated design mitigates immortal-time bias but cannot achieve the counterfactual purity of a randomized trial; nonetheless, sensitivity analyses were uniformly supportive. Second, although the cohort spanned multiple scanners and included harmonization, this remains a multicenter study with modest size; external validation on independent, prospectively accrued datasets is essential. Third, while we incorporated peripheral immune metrics (CBC, cytokines, flow), these are indirect surrogates for the intratumoral TIME. Future iterations should fuse MRI radiomics with on-treatment plasma proteomics, ctDNA, or myeloid single-cell states, and—where feasible—spatial transcriptomics to refine biological anchoring. Fourth, we did not capture structured patient-reported outcomes, psychosocial measures, or objective adherence metrics, which may influence real-world effectiveness and tolerability. Prior psycho-oncology work indicates that patient–oncologist alliance can affect adherence in advanced cancer settings (43). Future prospective validation should integrate adherence monitoring (e.g., dose interruptions, medication possession measures) and PRO instruments assessing symptom burden, function, mood, and quality of life; this is particularly relevant in glioma survivorship where neuropsychiatric outcomes may meaningfully shape treatment continuation and benefit. Fifth, although our explainability analyses localized attention to enhancing rims for benefit-positive cases, saliency approaches are model-dependent and can be sensitive to preprocessing (44); prospective reader-in-the-loop audit and pre-registration of analysis plans will improve reliability.

## 5 Conclusions

In a real-world HGG cohort, a pre-specified Radiomics–Immune Score identified a subgroup with clinically meaningful PFS benefit from CDK6 inhibitors without excess toxicity. The signal was robust across sensitivity analyses, well-calibrated, and demonstrated decision-analytic utility. These data support biomarker-guided application of CDK6 blockade in HGG and motivate prospective, enriched trials to confirm benefit and enable rational combinations.

## Data Availability

The de-identified clinical molecular testing reports and associated structured metadata available for this study are deposited under controlled access in the Open Archive for Miscellaneous Data (OMIX), National Genomics Data Center, China National Center for Bioinformation / Beijing Institute of Genomics, Chinese Academy of Sciences, accession number OMIX020548, with associated BioProject accession PRJCA074200. Access requests are reviewed by the data submitter and the responsible institution in accordance with participant consent, institutional approval, privacy safeguards, and applicable Chinese human genetic resource requirements.
